# RANKL blockade alleviates peri-implant bone loss and is enhanced by anti-inflammatory microRNA-146a through TLR2/4 signaling

**DOI:** 10.1186/s40729-020-00210-0

**Published:** 2020-04-15

**Authors:** Keqing Pan, Yang Hu, Yufeng Wang, Hao Li, Michele Patel, Danyang Wang, Zuomin Wang, Xiaozhe Han

**Affiliations:** 1 Department of Stomatology, The Affiliated Hospital of Qingdao University, College of Stomatology, Qingdao University, Qingdao, 266003 Shandong China; 2 Department of Immunology and Infectious Diseases, The Forsyth Institute, 245 First Street, Cambridge, MA 02142 USA; 3 Department of Oral Medicine, Ninth People’s Hospital, National Clinical Research Center of Stomatology, , Shanghai Jiao Tong University School of Medicine; Shanghai Key Laboratory of Stomatology & Shanghai Research Institute of Stomatology, Shanghai, 200011 China; 4 Department of Prosthodontics, The Affiliated Hospital of Stomatology, Guangxi Medical University, Nanning, 530021 China; 5 Department of Stomatology, Beijing ChaoYang Hospital affiliated with Capital Medical University, Beijing, China

**Keywords:** RANKL, Bone resorption, Peri-implantitis, MiR-146a, TLR2, TLR4

## Abstract

**Background:**

The present study was to determine the effect of local anti-RANKL antibody administration in the presence or absence of microRNA-146a on ligature-induced peri-implant bone resorption, and the potential role of TLR2/4 signaling in such effect.

**Results:**

Titanium implants were placed in the left maxilla alveolar bone 6 weeks after extraction of first and second molars in C57/BL6 wild-type (WT) and TLR2^−/−^ TLR4^−/−^ (TLR2/4 KO) mice. Silk ligatures were tied around the implants 4 weeks after implantation. Anti-RANKL antibody (500 μg/mL) with or without microRNA 146a (miR-146a) (100 nM) was injected into palatal gingiva around implant on days 3, 6, and 9 during 2 weeks of ligation period. Bone resorption around the implants was assessed by 2D imaging using area measurement and 3D imaging using micro-computed tomography (μCT). Real-time quantitative PCR (RT-qPCR) was used to determine the peri-implant gingival mRNA expression levels of pro-inflammatory cytokines (TNF-α) and osteoclastogenesis-related cytokines (RANKL). In both WT and TLR2/4 KO mice, the bone resorption around implants was significantly increased in the ligation only group when compared to the non-ligation group, but TLR2/4 KO mice showed significantly less bone loss compared to WT mice after ligation. As expected, gingival injection of anti-RANKL antibody significantly reduced bone loss compared with the ligation only group in both WT and TLR2/4 KO mice. Moreover, injection of miR-146a in addition to anti-RANKL antibody significantly enhanced the inhibition of bone loss in WT mice but not in TLR2/4 KO mice. Gingival mRNA expressions of RANKL were significantly reduced by anti-RANKL antibody treatment in both WT and TLR2/4 KO mice but were not affected by the additional miR-146a treatment. Gingival mRNA expression of TNF-α was significantly reduced by miR-146a treatment in WT mice but not in TLR2/4 KO mice. The number of gingival inflammatory cell infiltration and peri-implant TRAP-positive cell formation was significantly reduced by the additional miR-146a treatment in WT mice but not in TLR2/4 KO mice.

**Conclusions:**

This study suggests that anti-inflammatory miR-146a enhance anti-RANKL-induced inhibition of peri-implant bone resorption through the regulation of TLR2/4 signaling and inhibition of TNF-α expression.

## Background

Dental implant has become a preferable choice to restore the missing tooth in the past few decades for functional and esthetic purposes [1]. However, peri-implantitis has become prevalent accompanying the exponential growth of dental implant procedures [2, 3]. Peri-implantitis is indicated by infection of implant surrounding soft tissues and bone loss, resulting in implant failure eventually [4–6]. The host immune and inflammatory responses caused by plaques on implant surface are crucial in the pathogenesis of peri-implantitis [2, 7, 8]. However, current treatment available for peri-implantitis is not satisfactory due to the lack of understanding of the mechanism of peri-implantitis pathogenesis.

Receptor activator of nuclear factor-kappa B (RANK) and its ligand RANKL and the decoy receptor osteoprotegerin (OPG) are central regulators of osteoclast development and essential for osteoimmunology [9–12]. Recent study showed that RANKL/OPG ratio was significantly increased in the gingival tissues surrounding mini-implants in the rat model with *Porphyromonas gingivalis* LPS inductions [13]. Moreover, anti-RANKL antibody was approved for the treatment of osteoporosis, and it showed inhibition of bone loss in rodent experimental periodontitis models [14–17]. Our previous study showed that administration of anti-RANKL antibody directly to the gingival of rat experimental periodontitis model can significantly reduce gingival sRANKL expression and of bone resorption [18]. However, the effects of anti-RANKL antibody on peri-implantitis have not been investigated.

MicroRNAs (miRs) are small non-coding RNA molecules found in plants, animals, and some viruses, functioning in RNA silencing and post-transcriptional regulation of gene expression [19–21]. Recent studies showed that miRs are important regulators in periodontitis [21–23]. Our previous studies demonstrated that miR-146a regulated the cytokine secretion in human gingival fibroblasts and periodontal ligament cells and inhibits inflammatory cytokine production in B cells through directly targeting IRAK1, suggesting a regulatory role of miR-146a in immune-mediated periodontal inflammation [24]. However, the role of miR-146a in peri-implantitis remains unknown.

Toll-like receptors (TLR) are a family of well-characterized pattern recognition receptors (PRRs) and play an important role in the induction of pro-inflammatory cytokines by recognizing the signature molecules of the host innate immunity [25–27]. Our previous studies showed that TLR2 are associated with implant bone loss in a mouse model of peri-implantitis [5] and TLR4 is essential for periodontal bone loss [28, 29]. In our previous study, we examined the changes of inflammatory cytokines and bone metabolism cytokines in either TLR2 only KO mice or TLR4 only KO mice [5, 29]. However, since anti-RANKL antibody and miR-146a may interact with both TLR2 and TLR4 pathways, TLR2 and TLR4 double knockout (TLR2/4 KO) mice were specially employed in the present study to determine whether the effects of local anti-RANKL antibody administration in the presence or absence of miR-146a on ligature-induced peri-implant bone loss are dependent on both TLR2 and TLR4.

While our previous studies have substantiated that RANKL blockade inhibited immune-mediated RANKL-dependent bone loss, others have indicated that proinflammatory cytokines, such as SOFAT and TNF-alpha, could induce osteoclastogenesis in a RANKL-independent manner [30–32] through TLR signaling pathway [33]. MiR-146 has been implicated in the involvement of the innate immune responses through negative feedback regulation of TLR signaling [34]. In particular, recent studies have concluded that miR-146a has a diverse and critical role in limiting an excessive acute inflammatory reaction [35]. The purpose of the current study is to investigate the potential synergistic effect of RANKL blockage and anti-inflammatory miR-146a in the control of peri-implant bone loss. Our hypothesis is that anti-inflammatory microRNA-146a synergistically enhance anti-RANKL antibody-induced inhibition of peri-implant bone loss through TLR2/4 signaling.

## Methods

### Mice

Wild-type (WT) C57BL/6 and TLR2 KO and TLR4 KO mice in C57/BL6 background were purchased from the Jackson Laboratory (Bar Harbor, ME). TLR2 and TLR4 double KO mice (TLR2/4 KO) were crossbreed from TLR2 KO and TLR4 KO mice and confirmed by genotyping. All the animal-associated protocols were reviewed and approved (#17-022) by the Institutional Animal Care and Use Committee of the Forsyth Institute. All the mice used in the study were maintained in specific pathogen-free units. Mice were fed a soft diet ad libitum for the duration of the experiment. Forty-eight WT mice and forty-eight TLR2/4 KO mice were used in this study, and the mice were randomly divided into 4 groups in WT and TLR2/4 KO mice as follows: (1) implant only (*n* = 12), (2) implant + ligation (*n* = 12), (3) implant + ligation+ anti-RANKL antibody (*n* = 12), and (4) implant + ligation + anti-RANKL antibody + miR-146a (*n* = 12).

### Tooth extraction, implant placement, and ligature-induced experimental peri-implantitis and local administration of anti-RANKL-antibody and miR-146a

The procedures of tooth extraction and implant placement were as previously described [5]. Briefly, all the mice had their left maxillary first and second molars extracted at 4 weeks old with 6 weeks of healing time after the tooth extraction. Drinking water with antibiotics (sulfamethoxazole and trimethoprim, 850 μg/170 μg per mL) was used for 2 weeks to decrease the possibility of infection after tooth extraction. Then, the maxillary alveolar bone was drilled with the 0.3-mm-diameter carbide micro-hand drill, and a smooth-surface, screw-shaped titanium implant (1 mm in length and 0.5 mm in diameter, D. P. Machining) was screwed clockwise into the bone through the drilled site until torque could be achieved. Implants were allowed to heal for 4 weeks, and the antibiotic water was given during the first week after implantation as described above. Four weeks after implant placement, experimental peri-implantitis was initiated with a 7-0 silk ligature tied around the implants. The ligation day was recorded as day 0, and the ligature remained in place for 2 weeks. For group 3 and group 4, each mouse received palatal gingival injections of 2 μL of anti-RANKL antibody (500 μg/mL, Peprotech, Rocky Hill, NJ) or miR-146a (100 nM, GeneCopoeia, Rockville, MD) on the mesial and distal gingival papillae of implant by a 31-gauge double-beveled MicroFine needle (Becton, Dickinson) as these doses were established from our previous publications [24, 28]. The injections for animals were administered three times on days 3, 6, and 9, and all the mice were euthanized by CO_2_ inhalation on day 14. All the procedures, including tooth extraction, implant placement, ligature placement, and injection, were performed using an optical microscope (S6D Stereozoom, Leica).

### Tissue collection and sample preparation

The ligations were maintained for 2 weeks, and after which the mice were euthanized by CO_2_ inhalation and the maxilla were harvested. The gingival tissues of half group of mice were isolated and collected for mRNA expression study. The skulls left were defleshed by beetles for 1 week. Briefly, in beetle’s chamber, freshly dissected skull was put in a paper cup with 0.5 cm diameter holes at the bottom, so beetles can move in with relatively controlled numbers. After that, the skulls were bleached by H_2_O_2_ (3%) for 4 h. Bone resorption was measured by microscope imaging analysis and μCT scan analysis. The skulls of the other half of the group were fixed in formalin overnight at 4 °C followed by EDTA decalcification for 3 weeks with agitation. After complete demineralization, implants were removed manually by rotating counterclockwise. All the decalcification samples were embedded into paraffin and cut in 5 μm sections along the mesial-distal plane and then subjected to H&E staining and TRAP staining.

### Imaging analysis of bone resorption area

The two dimension (2D) bone resorption measurements were assessed under a microscope (Nikon SMZ745T, Nikon Instruments Inc.) and analyzed by software ImageJ (NIH) on buccal and palatal surfaces for each segment, and a standard calibrator was used for calibration at the same magnification as previously described [36]. The bone resorption area was enclosed coronally by the CEJ of the molars, laterally by the exposed distal root of the first molar and the exposed mesial root of the third molar, and apically by the alveolar crest. The results are presented in square millimeters.

### Micro-computed tomography analysis

Mice maxillae were scanned with a high-resolution scanner (mCT-40, Scanco Medical). Samples were exposed to polychromatic X-rays on a rotating stage at a steep angle of 0.18° over 360°. Measurements were taken at an operating voltage of 70 kVp and 114 mA current and 6 mm isotropic voxel resolution, with an exposure time of 200 ms and five frames averaged per view. Quantitative three dimension (3D) measurements of the bone resorption were performed using Seg3D software as previously described [5].

### Real-time quantitative PCR

Palatal gingival tissues were isolated from around ligatured implants and were homogenized in lysis buffer using a tissue homogenizer. Total RNA was extracted using PureLink® RNA Mini Kit (Ambion). cDNA was synthesized using the SuperScript III Reversed Transcriptase kit (Invitrogen) according to the manufacturer’s protocol. The mRNA expression of TNF-α and RANKL in gingival was determined by real-time quantitative PCR (RT-qPCR) using LightCycler® SYBR Green I master and LightCycler® 480 Instrument system (Roche). GAPDH gene was used as an internal control. The sequences of primers are listed as follows: TNF-α forward 5′-CACAGAAAGCATGATCCGCGACGT-3′; TNF-α reverse 5′-CGGCAGAGAGGAGGTTGACTTTCT-3′; RANKL forward 5′-GGGTGTGTACAAGACCC-3′; RANKL reverse 5′-CATGTGCCACTGAGAACCTTGAA-3″ GAPDH forward 5′-CCCCAGCAAGGACACTGAGCAA-3′; GAPDH reverse 5′-GTGGGTGCAGCGAACTTTATTGATG-3′.

### Hematoxylin and eosin staining and tartrate-resistant acid phosphatase staining

All the maxilla collected were fixed in 4% formaldehyde overnight and then went through decalcification in 10% EDTA for 3 weeks at 4 °C with shaking. Five-micrometer-thick sections were produced in the mesial-distal plane for hematoxylin and eosin (H&E) and tartrate-resistant acid phosphatase (TRAP) staining. An acid phosphatase kit (catalog number 387A, Sigma) was used for TRAP staining. After 30 min staining and 1 min counterstain with hematoxylin, TRAP-positive cells with three or more nuclei were considered to be osteoclasts. A region of interest (ROI) was defined in peri-implantitis samples as a 1.5 × 1-mm rectangular area aligned with the central long axis of the implant and covered the whole length of the implant. TRAP-positive cell numbers within the ROI were quantified manually by ImageJ. For H&E staining, images were analyzed by ImageJ after being captured by a digital camera. The numbers of inflammatory cells from the implant supportive tissue on each slide were counted at a magnification of × 40, and the average numbers were calculated.

### Statistical analysis

Results were presented as mean ± SD. Unpaired Student’s *t* test was used to analyze differences between any two groups of data sets. Results with *p* < 0.05 are considered statistically significant.

## Results

### Ligature-induced peri-implant inflammation and bone loss in mice

**Fig. 1:**
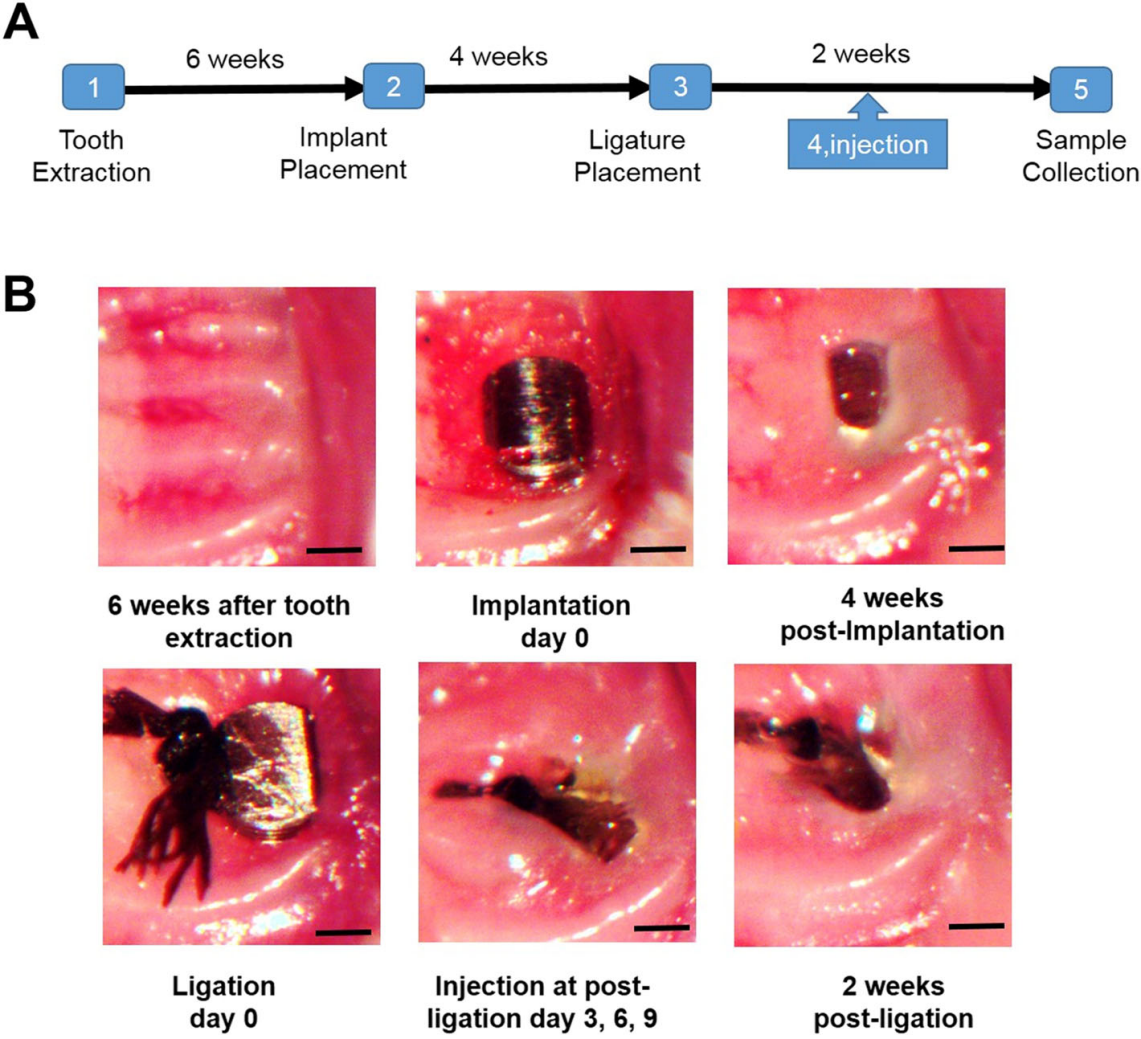
Mouse model of ligature-induced experimental peri-implantitis. (**a**) Tooth extraction: left maxillary first and second molars extracted at 4 weeks old and the tooth extraction socket healed well with smooth gingiva surface after 6 weeks post-extraction. Implant placement: implant was put in alveolar bone without flap elevation. Ligature placement: at 4 weeks post-implant, 7-0 ligatures were applied under the fixture head. Gingival injection: injections for animals were administered three times on days 3, 6, and 9 during 14 days ligation period. Sample collection: 14 days post-ligation, the gingival tissues and the skulls were collected. (**b**) Images depicting processing steps of the experimental design (scale bar, 500 μm)

**Fig. 2:**
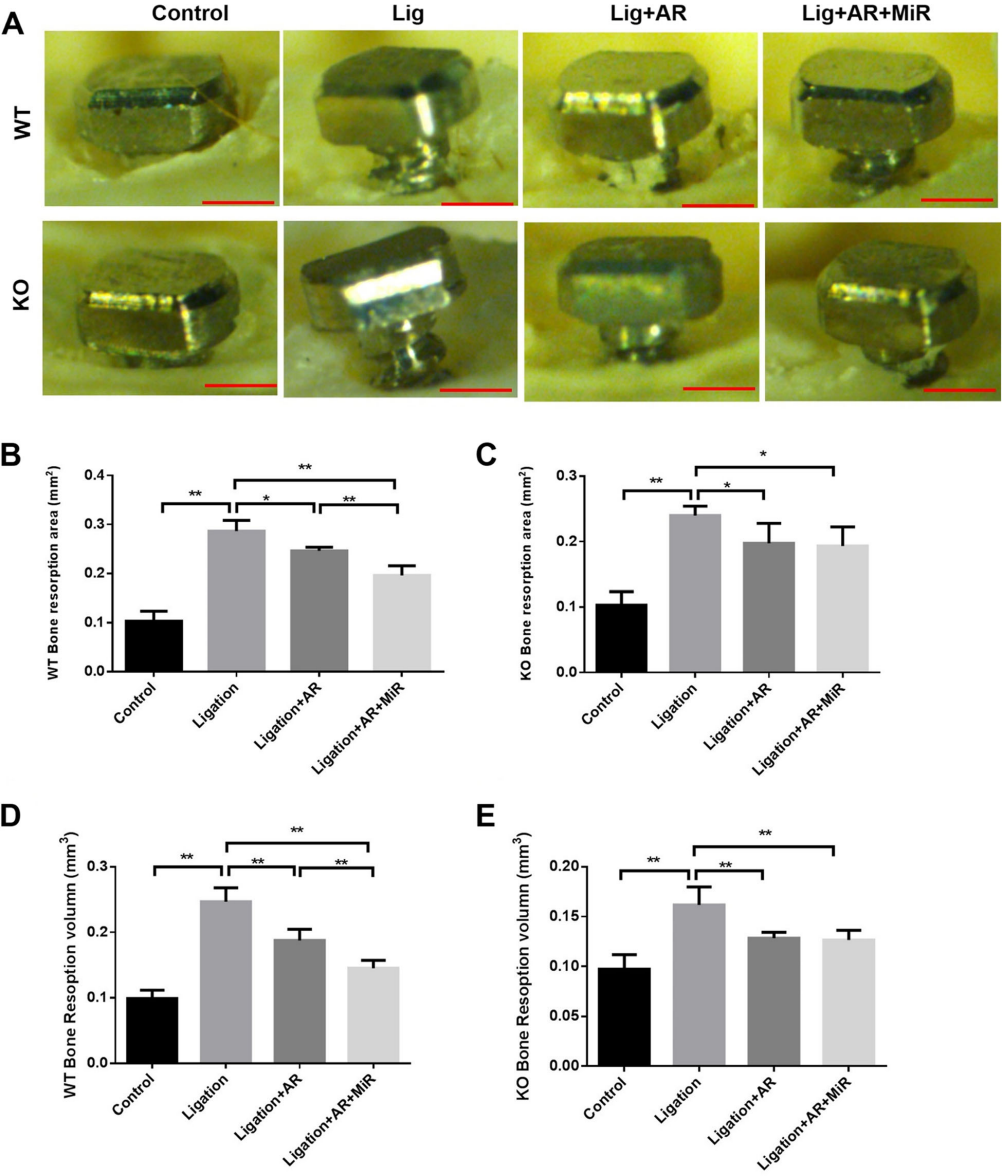
Anti-RANKL and anti-RANKL+miR-146a treatments decreased ligature-induced bone resorption with different patterns in experimental peri-implantitis of WT and TLR2/4 KO mice. Buccal side images of the defleshed skulls were taken of the control (non-ligation) group, ligation (non-treatment) group, ligation with anti-RANKL antibody (ligation+AR) treatment group, and ligation with anti-RANKL antibody + miR-146a (ligation+A+MiR) treatment group in WT mice and TLR2/4 KO mice (**a**) (scale bar, 500 μm). The bone resorption area based on these images was measured and analyzed for WT mice (**b**) and TLR2/4 KO mice (**c**) (mean ± SD, *n* = 6, *p < 0.05, **p < 0.01, SEM, standard error of difference between two means). Three dimension (3D) images from μCT were collected and analyzed for WT mice (**d**) and TLR4 KO mice (**e**) (mean ± SD, *n* = 6, *p < 0.05, **p < 0.01)

In our ligature-induced experimental peri-implantitis mouse model, teeth extraction, implant placement, ligation placement, and gingival injection will be performed in a 12-week process (Fig. 1). 86.67% (52 out of 60) of implants in TLR2/4 KO mice achieved osteointegration (no mobility when touched by needles, no obvious bleeding upon probing) after being placed for 4 weeks, which has no significant difference with WT mice success rate 81.67% (Table 1). The gingival tissue surrounding the implant appeared healthy (pink in color, no obvious swelling, no bleeding upon probing) 28 days after implant placement in WT and TLR2/4 KO mice, but gingival tissue turned red in color and displayed apparent swelling 3 days after the ligature was placed. Furthermore, the ligation group showed a significantly higher bone resorption compared with the non-ligation group in WT mice, for both 2D imaging analysis (*p* = 0.0021, Fig. 2a, b) and μCT analysis (*p* = 0.0012, Fig. 2d), and in TLR2/4 KO mice for both 2D imaging analysis (*p* = 0.0039, Fig. 2a, c) and μCT analysis (*p* = 0.0023, Fig. 2e). Taken together, these results indicate that ligature successfully induced inflammation and bone resorption in this mouse model of peri-implantitis.

**Table 1:** Success rate (SR) of osseointegrated implants 4 weeks after implant placement

	Total implants	Lost	Loose	Osseointegrated	Success rate (%)	SR *P* value
Wild type group	60	6	5	49	81.67	0.595
TLR2/4 KO group	60	4	4	52	86.67

### Anti-RANKL antibody and anti-RANKL+miR-146a treatments showed different effects on peri-implantitis bone loss in WT and TLR2/4 KO mice

**Fig. 3:**
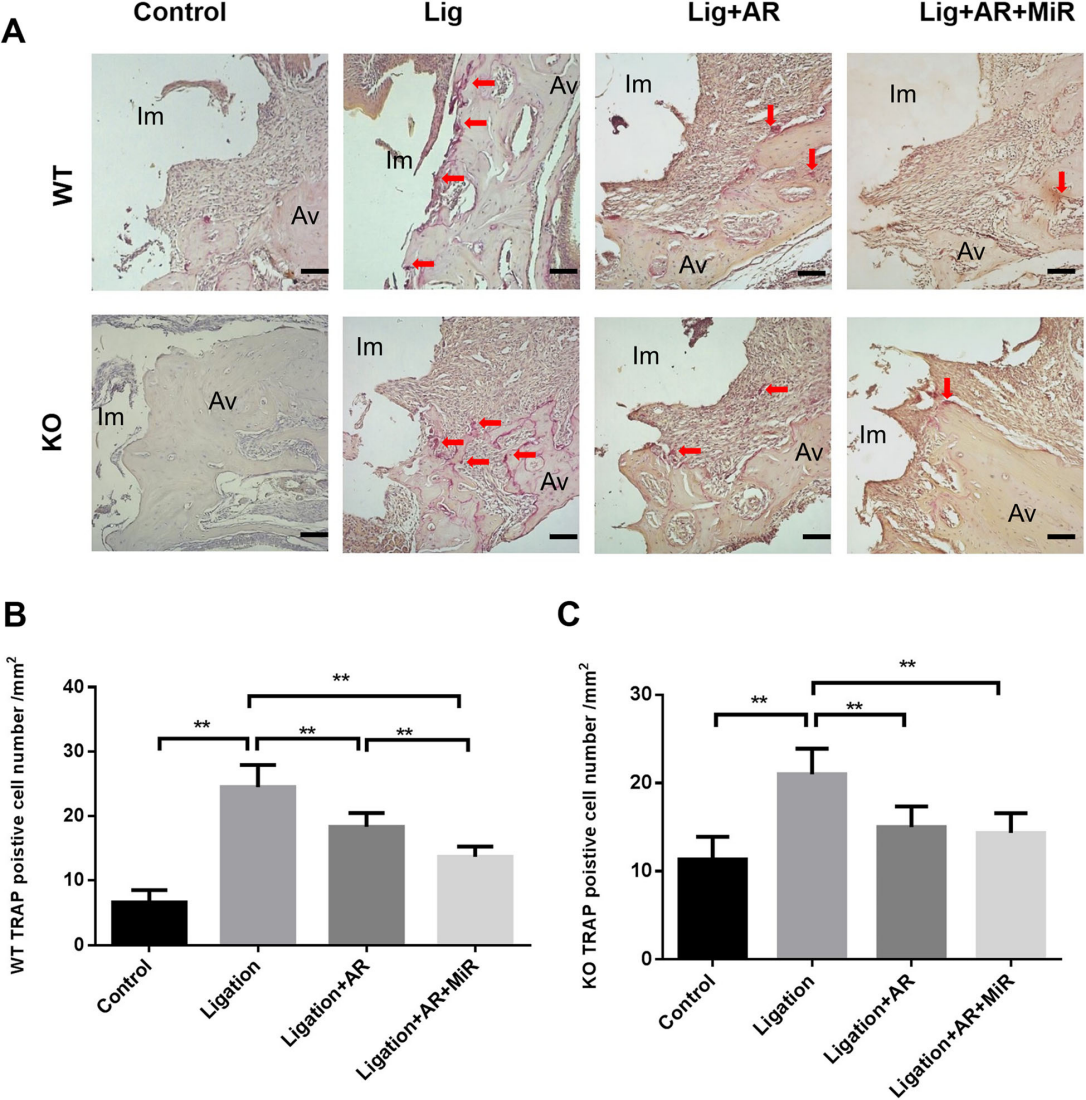
Anti-RANKL and anti-RANKL+miR-146a treatments decreased TRAP-positive cell quantities with different patterns in experimental peri-implantitis of WT and TLR2/4 KO mice. TRAP-positive cells (red color) with 3 or more nuclei were considered osteoclasts and were shown in the control group, ligation group, ligation with anti-RANKL antibody treatment group, and ligation with anti-RANKL antibody + miR-146a treatment group in WT mice and TLR2/4 KO mice (**a**) (Im, implant; Av, alveolar bone; scale bar, 100 μm). The quantities of TRAP-positive cells were analyzed in each group of WT mice (**b**) and TLR2/4 KO mice (**c**) (mean ± SD, *n* = 6, **p < 0.01)

Anti-RANKL antibody alone significantly reduced bone loss compared with the ligation only group in both WT and TLR2/4 KO mice; however, injection of miR-146a in addition to anti-RANKL antibody significantly enhanced the inhibition of bone loss in WT mice but not in TLR2/4 KO mice (Fig. 2b–e). Significantly higher number of osteoclasts were observed in the ligation group vs. non-ligation group in WT mice and TLR2/4 KO mice in TRAP staining (Fig. 3a–c), which is consistent with the results of 2D and 3D bone loss analysis (Fig. 2b–e). Moreover, the anti-RANKL+miR-146a treatment groups showed significantly lower number of osteoclasts compared with the anti-RANKL treatment group in WT mice but not in TLR2/4 KO mice (Fig. 3b, c), which is also consistent with the results of bone loss analysis (Fig. 2b–e). Taken together, miR-146a in addition to anti-RANKL antibody can further reduce bone loss in WT mice but is ineffective when TLR2 and TLR4 are deficient, suggesting miR-146a anti-bone loss effects in peri-implantitis are TLR2/4 dependent.

### Anti-RANKL antibody and anti-RANKL+miR-146a treatments had different effects on peri-implantitis inflammation in WT and TLR2/4 KO mice

**Fig. 4:**
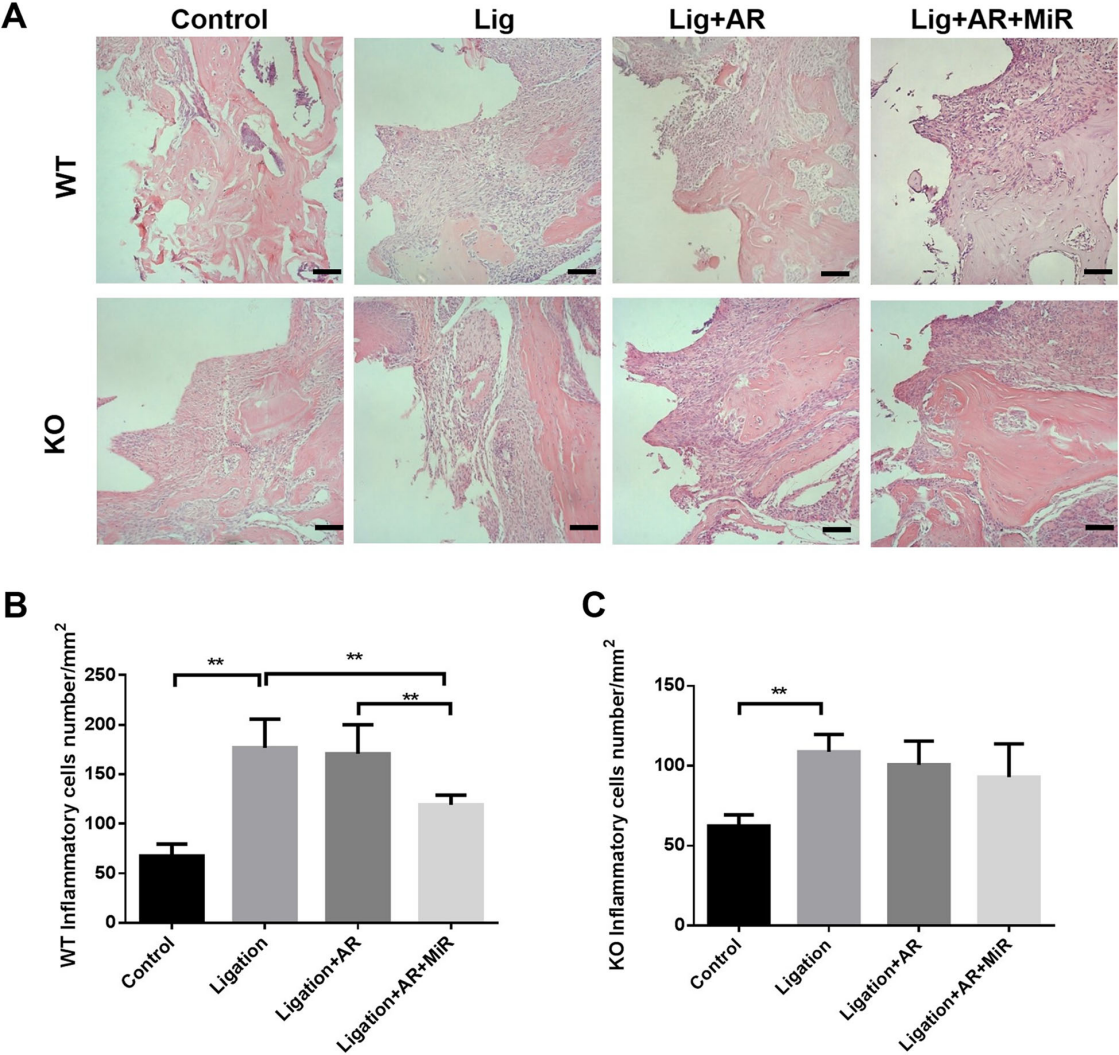
Anti-RANKL and anti-RANKL+miR-146a treatments decreased the inflammatory cell infiltration of the implant gingival tissues with different patterns in experimental peri-implantitis of WT and TLR2/4 KO mice. HE staining of the gingival tissue around implants were performed in the control group, ligation group, ligation with anti-RANKL antibody treatment group, and ligation with anti-RANKL antibody + miR-146a treatment group in WT mice and TLR2/4 KO mice (**a**) (scale bar, 100 μm). Inflammatory cell numbers were measured and analyzed in each group of WT mice (**b**) and TLR2/4 KO mice (**c**) (mean ± SD, *n* = 6, **p < 0.01)

In both WT mice and TLR2/4 KO mice, a significantly higher number of inflammatory cells were found infiltrating around the peri-implant tissues in the ligation group compared with the non-ligation group (Fig. 4a–c). However, the number of inflammatory cells in tissues of the ligation group was not significantly changed when treated with anti-RANKL antibody alone in both WT and TLR2/4 KO mice compared with the ligation group (Fig. 4b, c). MiR-146a treatment additional to anti-RANKL antibody significantly decreased the number of inflammatory cells in WT mice but not in TLR2/4 KO mice when compared with the anti-RANKL antibody alone group. Taken together, anti-RANKL antibody alone did not affect inflammatory cell infiltration in peri-implantitis, and miR-146a showed anti-inflammatory effects only on WT mice but not on TLR2/4-deficient mice.

### Pro-inflammatory and bone metabolism factors showed different pattern of change when treated with anti-RANKL antibody and anti-RANKL+miR-146a in WT and TLR2/4 KO mice

**Fig. 5:**
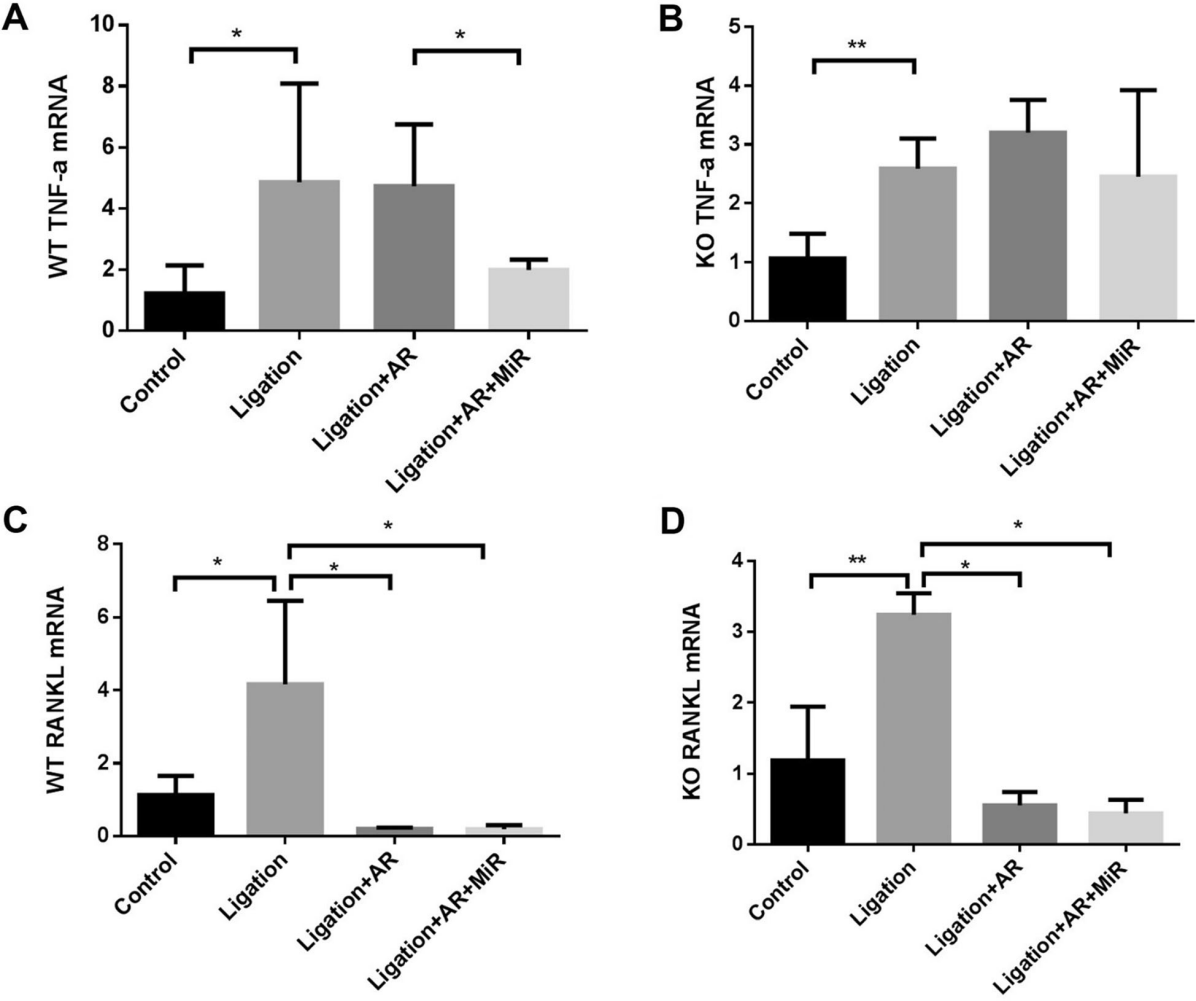
Anti-RANKL and anti-RANKL+miR-146a treatments decreased gingival mRNA expression of TNF-α and RANKL with different patterns in experimental peri-implantitis of WT and TLR2/4 KO mice. Gingival tissues around ligatured implants and non-ligation implants were excised and processed for RT-qPCR analysis to determine mRNA level of TNF-α of WT mice (**a**) and TLR2/4 KO mice (**b**) (mean ± SD, *n* = 6, *p < 0.05, **p < 0.01) and mRNA level of RANKL of WT mice (**c**) and TLR2/4 KO mice (**d**) (mean ± SD, *n* = 6, *p < 0.05, **p < 0.01).

Gingival TNF-α mRNA showed a significant upregulation in the ligation group compared with the non-ligation group in both WT and TLR2/4 KO mice (Fig. 5a, b). Moreover, TNF-α showed no significant decrease when treated with anti-RANKL antibody alone in both WT and TLR2/4 KO mice compared with the ligation group. However, additional miR-146a treatment significantly decreased TNF-α expression in WT mice but not in TLR2/4 KO mice (Fig. 5a, b), suggesting the consistent results of anti-inflammation effects characterized by quantification of infiltrating inflammation cells (Fig. 4a–c). Meanwhile, gingival RANKL mRNA expression was significantly decreased with anti-RANKL antibody alone in both WT and TLR2/4 KO mice, and additional miR-146a treatment did not show significant difference compared with the anti-RANKL antibody treatment group (Fig. 5c, d). Taken together, anti-RANKL antibody showed the inhibition effects on RANKL expression in both WT and TLR2/4 KOTLR2/4 KO mice, and miR-146a showed anti-inflammation effect through downregulation of TNF-α mRNA only in WT mice.

## Discussion

Our present study showed that anti-RANKL antibody can significantly inhibit the bone loss in peri-implantitis and additional miR-146a treatment will enhance this inhibition through its anti-inflammation effects via TLR2/4 signaling. This is the first report in a murine model of peri-implantitis to demonstrate that anti-RANKL antibody and miR-146a together can significantly reduce bone resorption and inflammation in peri-implantitis, suggesting a potential therapeutic strategy for peri-implantitis patients. Moreover, the data showed that anti-bone loss effects of anti-RANKL antibody are independent of TLR2 and TLR4 and anti-RANKL antibody alone did not affect peri-implant inflammatory infiltration, suggesting that RANKL modulation of bone loss is downstream of peri-implant inflammation and more directly towards bone resorption. However, anti-RANKL antibody + miR-146a treatment showed significantly stronger inhibition of bone loss than anti-RANKL antibody alone treatment, indicating that the suppression of inflammation can be used to reduce peri-implantitis bone loss by removing additional RANKL-independent etiology and pathogenesis of bone loss. These data (Fig. 1–5, Supplemental Table S1) showed that anti-inflammatory miR-146a enhance anti-RANKL-induced inhibition of peri-implant bone resorption through the regulation of TLR2/4 signaling and inhibition of TNF-α expression. Thus, the ideal osteoimmunological treatment for peri-implantitis should include both direct anti-osteoclastogenesis and anti-inflammation components.

As innate immune recognition receptors, TLR family plays a central role in innate immunity, inflammation, cell survival, and proliferation [5, 26, 37, 38]. TLR2 and TLR4 are essential signaling proteins in progression of inflammation and related bone metabolism in periodontitis [28, 29, 39, 40]. However, little is known about the functions of TLR2 and TLR4 signaling in peri-implantitis. In the present study, we investigated the changes in bone resorption, gingival TNF-α (pro-inflammatory marker) mRNA levels, and gingival soluble RANKL protein levels in ligation-induced experimental peri-implantitis in WT and TLR2/4 KO mice with or without anti-RANKL antibody alone treatment and anti-RANKL antibody + miR-146a treatment. The results showed that anti-bone loss effects of anti-RANKL antibody are TLR2/4 independent but anti-inflammation and related anti-bone loss effects of miR-146a are TLR2/4 dependent, suggesting that TLR2/4 signaling is crucial for RANKL-independent, inflammation-induced bone loss in peri-implantitis.

According to the previous studies, miR-146a was regulated by NF-κB and blockade of miR-146a could decrease TLR4 and NF-κB in human cells [41, 42], suggesting that miR-146a is involved in TLR/NF-κB signaling pathway. Our recent study showed that miR-146a inhibited inflammatory cytokine secretion in B cells after challenged with *P. gingivalis* LPS and decreased bone resorption in experimental periodontits animal models [24]. Moreover, it was found that miR-146a negatively regulated TLR2-induced inflammatory response in keratinocytes [43] and expression of TLR2 was repressed by miR-146a in HEK293T cells [44]. Thus, the cross talk between miR-146a and TLR2/4 may be essential for anti-inflammation effects of miR-146a by inhibiting NF-κB signaling. In the present study, the data showed that miR-146a have no effects on inflammatory cell infiltration or TNF-α expression in the absence of TLR2 and TLR4 in experimental peri-implantitis, suggesting that miR-146a anti-inflammation effects are TLR2/4 dependent in peri-implantitis. However, on the other hand, how induction of TLR2/4 in oral disease affects the expression and function of miR-146a may need further investigation.

## Conclusions

In summary, the present study suggests that anti-inflammatory miR-146a enhance anti-RANKL-induced inhibition of peri-implant bone resorption through the regulation of TLR2/4 signaling and inhibition of TNF-α expression. Combination of regimens antagonizing both osteoclastogenesis and inflammation may become a more effective strategy to ameliorate peri-implantitis bone loss.

## Supplementary information

**Additional file 1: Supplemental Table S1.** The numerical data of all graphs.

## Data Availability

Presented in the main paper

## Abbreviations

RANKL: Receptor activator of nuclear factor-kappa Β ligand
TLR: Toll-like receptor
WT: Wild type
miR-146a: MicroRNA 146a
μCT: Micro-computed tomography
RT-qPCR: Real-time quantitative PCR
TNF-α: Tumor necrosis factor alpha
OPG: Osteoprotegerin
LPS: Lipopolysaccharides
H&E: Hematoxylin and eosin
TRAP: Tartrate-resistant acid phosphatase
