# Supplementary material for: RANKL blockade alleviates peri-implant bone loss and is enhanced by anti-inflammatory microRNA-146a through TLR2/4 signaling
Source: Int J Implant Dent. 2020 Apr 15;6:15. doi: 10.1186/s40729-020-00210-0 (PMC7156533; doi:10.1186/s40729-020-00210-0)
Supplement: Supplementary file 1 — Additional file 1: Supplemental Table S1. The numerical data of all graphs. [file 40729_2020_210_MOESM1_ESM.docx]

**Supplemental Table.1** The numerical data of all graphs.

| Graph # | Groups | Values |
| --- | --- | --- |
| Figure 2B | Control | 0.1032 ± 0.009144 |
|  | Ligation | 0.2865 ± 0.01089 |
|  | Ligation+AR | 0.2460 ± 0.003742 |
|  | Ligation+AR+MiR | 0.1960 ± 0.01125 |
| Figure 2C | Control | 0.1032 ± 0.009144 |
|  | Ligation | 0.2400 ± 0.007055 |
|  | Ligation+AR | 0.1975 ± 0.01520 |
|  | Ligation+AR+MiR | 0.1931 ± 0.01313 |
| Figure 2D | Control | 0.0991 ± 0.005258 |
|  | Ligation | 0.2469 ± 0.008712 |
|  | Ligation+AR | 0.1877 ± 0.006903 |
|  | Ligation+AR+MiR | 0.1451 ± 0.004959 |
| Figure 2E | Control | 0.09727 ± 0.005941 |
|  | Ligation | 0.1618 ± 0.007400 |
|  | Ligation+AR | 0.1284 ± 0.002348 |
|  | Ligation+AR+MiR | 0.1266 ± 0.004013 |
|  |  |  |
| Figure3B | Control | 6.667 ± 0.7601 |
|  | Ligation | 24.50 ± 1.408 |
|  | Ligation+AR | 18.33 ± 0.8819 |
|  | Ligation+AR+MiR | 13.67 ± 0.6667 |
| Figure 3C | Control | 11.33 ± 1.054 |
|  | Ligation | 21.00 ± 1.183 |
|  | Ligation+AR | 15.00 ± 0.9661 |
|  | Ligation+AR+MiR | 14.33 ± 0.9189 |
|  |  |  |
| Figure 4B | Control | 67.50 ± 4.958 |
|  | Ligation | 176.7 ± 11.88 |
|  | Ligation+AR | 170.7 ± 12.04 |
|  | Ligation+AR+MiR | 119.2 ± 3.936 |
| Figure 4C | Control | 62.17 ± 2.892 |
|  | Ligation | 108.7 ± 4.447 |
|  | Ligation+AR | 100.5 ± 6.081 |
|  | Ligation+AR+MiR | 92.83 ± 8.507 |
|  |  |  |
| Figure 5A | Control | 1.230 ± 0.4102 5 |
|  | Ligation | 4.865 ± 1.315 |
|  | Ligation+AR | 4.730 ± 0.9049 |
|  | Ligation+AR+MiR | 1.992 ± 0.1533 |
| Figure 5B | Control | 1.066 ± 0.1857 |
|  | Ligation | 2.590 ± 0.2966 |
|  | Ligation+AR | 3.204 ± 0.3205 |
|  | Ligation+AR+MiR | 2.450 ± 0.8521 |
| Figure 5C | Control | 1.131 ± 0.2145 |
|  | Ligation | 4.165 ± 1.024 |
|  | Ligation+AR | 0.2044 ± 0.01650 |
|  | Ligation+AR+MiR | 0.1888 ± 0.05763 |
| Figure 5D | Control | 1.189 ± 0.3776 |
|  | Ligation | 3.243 ± 0.1520 |
|  | Ligation+AR | 0.5505 ± 0.09636 |
|  | Ligation+AR+MiR | 0.4376 ± 0.09660 |
